# Arthroscopic Treatment of Isolated Pectoralis Minor Syndrome After Two Unsuccessful Cervical Spine Procedures: A Case Report

**DOI:** 10.7759/cureus.114205

**Published:** 2026-08-09

**Authors:** Omar Valdez, Pedro Pelaez Damy, Victor A Orihuela Fuchs, Carlos Mangino Rivas, Rodrigo Anaya Eulogio

**Affiliations:** 1 Orthopaedics and Traumatology, Centro Médico ABC, Mexico City, MEX

**Keywords:** arthroscopic shoulder surgery, brachial plexus neuropathies, neurogenic thoracic outlet syndrome, pectoralis minor syndrome, scapular dyskinesis, scapular winging, suprascapular nerve

## Abstract

Pectoralis minor syndrome (PMS) is an uncommon cause of neurogenic thoracic outlet syndrome and is frequently overlooked because its clinical manifestations resemble cervical radiculopathy and other shoulder disorders. We report a case of a 67-year-old female who developed persistent upper-extremity pain and paresthesia despite undergoing two cervical spine procedures. Following comprehensive clinical evaluation, provocative testing, and brachial plexus magnetic resonance imaging, isolated PMS was diagnosed. The patient underwent arthroscopic pectoralis minor tenotomy combined with suprascapular nerve release and brachial plexus neurolysis, resulting in marked pain relief, functional recovery, and return to recreational activities after one year. This case highlights the importance of considering PMS in patients with persistent symptoms after cervical spine surgery and supports arthroscopic decompression as an effective treatment in carefully selected patients.

## Introduction

Pectoralis minor syndrome (PMS) is an underrecognized cause of neurogenic compression involving the brachial plexus beneath the pectoralis minor tendon. As its clinical manifestations frequently resemble cervical radiculopathy, rotator cuff disorders, or other causes of shoulder pain, establishing the diagnosis may be challenging and often requires exclusion of more common conditions. As a result, many patients experience prolonged symptoms before the underlying source of compression is identified [1].

Thoracic outlet syndrome (TOS) encompasses a group of disorders caused by compression of the neurovascular structures as they travel from the cervical region into the upper extremity. Neurogenic TOS (nTOS) accounts for most cases, whereas vascular forms are considerably less common [2,3].

Compression may occur at several anatomical locations, including the interscalene triangle, the costoclavicular interval, and the retropectoralis minor space [4]. Increasing clinical evidence suggests that isolated compression beneath the pectoralis minor tendon represents a distinct clinical entity rather than merely a subtype of TOS [5,6]. Patients with PMS often present with poorly localized pain, paresthesia, weakness, or activity-related symptoms that can fluctuate according to shoulder position. As no single imaging modality is diagnostic, evaluation relies primarily on a detailed history, targeted physical examination, provocative maneuvers, and selective use of complementary imaging or diagnostic injections when indicated [7,8].

We present a case of a patient whose symptoms persisted despite undergoing two cervical spine operations before the correct diagnosis of isolated PMS was established. This case highlights the importance of maintaining a broad differential diagnosis in patients with persistent upper-extremity symptoms and illustrates the role of arthroscopic decompression as a treatment option after failure of conservative management.

## Case presentation

A 67-year-old female presented to our institution with a two-year history of left upper extremity pain accompanied by numbness and paresthesia. She reported a fall approximately six months before symptom onset, although no significant injury was identified at that time. Before referral to our institution, cervical magnetic resonance imaging demonstrated multilevel degenerative disc disease associated with congenital cervical canal narrowing at C4-C5, C5-C6, and C6-C7, together with bilateral neural foraminal stenosis, predominantly on the left side. Based on these imaging findings and persistent radicular symptoms, she underwent an anterior cervical discectomy and fusion (ACDF) from C4 to C7. As her symptoms failed to improve, she subsequently underwent left-sided C6-C7 laminectomy, foraminotomy, and nerve root decompression. Despite technically successful cervical spine procedures, she continued to experience disabling shoulder pain, diffuse paresthesia involving the entire left hand, and dysesthesia over the posterior aspect of the shoulder, prompting further diagnostic evaluation.

Diagnostic assessment

Given the persistence of symptoms despite two appropriately indicated cervical spine procedures, a comprehensive shoulder and peripheral nerve evaluation was performed to investigate alternative causes of neurogenic pain. Anamnestic strategies involved a detailed patient history focusing on the onset, duration, and characteristics of symptoms. Additionally, a battery of clinical and instrumental tests was implemented to rule out other potential pathologies, and the diagnosis of nTOS gained further support from positive results in various provocative tests, including the Roos, Elvey, Cyriax, and Wright tests, whereas the Adson test was negative.

These tests helped guide our clinical reasoning process and contributed to the decision-making surrounding further investigations. Also, the range of motion was not limited in any plane; the Medical Research Council (MRC) muscle strength grading scale score was 4/5 due to pain and apprehension, primarily during abduction, flexion, and external rotation [9]. Physical examination demonstrated supraspinatus and infraspinatus muscle atrophy and scapular protraction, exacerbated by movement, thus presenting a picture of scapular dyskinesia. On palpation, there was hyperalgesia over the coracoid process and the anterior portion of the deltoid and pectoral muscles, as well as muscle contracture in the rhomboids and trapezius. Paresthesias were present throughout the hand, and dysesthesias were noted in the posterior region of the shoulder. The visual analog scale (VAS) showed a score of 9 at that time, and the patient reported this on most days. Disabilities of the Arm, Shoulder and Hand (DASH) and Constant-Murley scores were calculated, yielding 67.5% and 53%, respectively [10,11]. As the diagnosis of isolated PMS relies on the integration of clinical history, targeted physical examination, provocative maneuvers, and complementary imaging studies rather than on a single confirmatory test, the principal diagnostic features described in the literature are summarized in Table 1.

**Table 1:** Diagnostic approach to suspected isolated pectoralis minor syndrome. PMS: pectoralis minor syndrome; STIR: short tau inversion recovery; MRI: magnetic resonance imaging; EMG: electromyography; NCS: nerve conduction studies. The table is based on the current literature regarding the clinical evaluation of isolated PMS. Findings in the present patient are described in the Case Presentation.

Assessment	Typical finding	Diagnostic significance
History	Pain, paresthesia, symptoms aggravated by overhead activity	Raises suspicion for neurogenic compression
Inspection	Scapular protraction or dyskinesis, muscle atrophy	Suggests chronic dysfunction
Palpation	Tenderness over the coracoid process and pectoralis minor	Localizes the compression site
Wright test	Reproduction of symptoms	Suggestive of retropectoralis minor compression
Roos test	Pain or paresthesia during elevated arm stress	Supports neurogenic thoracic outlet syndrome
Elvey (Upper Limb Tension Test)	Neural tension symptoms	Indicates brachial plexus irritation
Cyriax test	Pain reproduced by coracoid compression	Supports PMS
Adson test	Usually negative in isolated PMS	Helps exclude scalene compression
Scalene compression test	Usually negative	Helps distinguish PMS from scalene syndrome
Cervical MRI	Degenerative disease or normal findings	Evaluates cervical pathology
Brachial plexus MRI	Increased STIR signal adjacent to the retropectoralis minor space	Supports diagnosis after excluding other causes
Ultrasound-guided pectoralis minor muscle block	Temporary symptom relief	Useful adjunct to support the diagnosis in selected patients
Electrodiagnostic studies (EMG/NCS)	May be normal or nonspecific	Useful to exclude other neuropathies but not diagnostic

**Figure 1:**
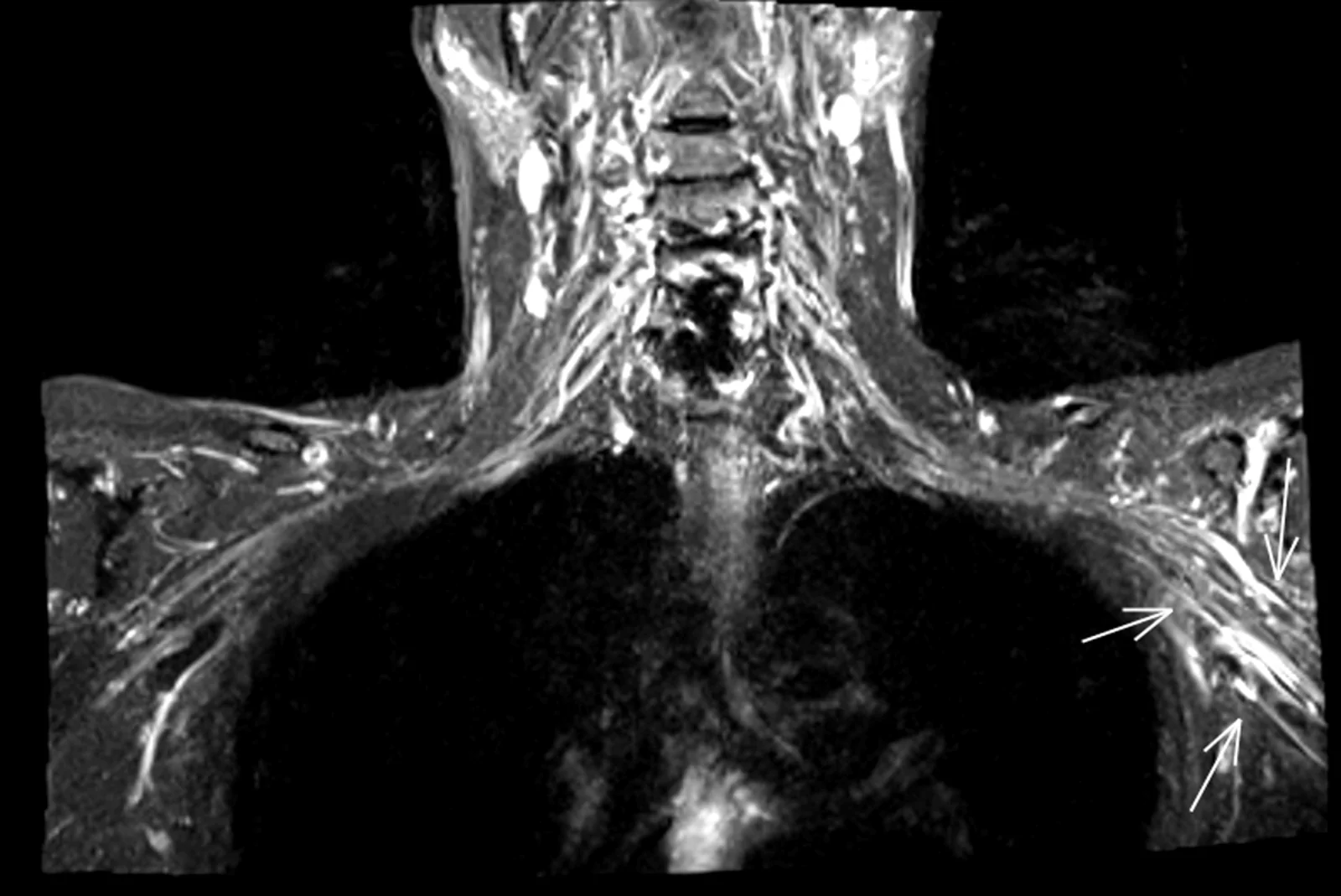
Coronal section of a brachial plexus MRI. The arrows show an increased signal from the proximal nerve tracts.

Magnetic resonance imaging of the brachial plexus was obtained on a 1.5-T Signa Artist magnetic resonance system (GE HealthCare, Chicago, IL). Short tau inversion recovery (STIR) sequences demonstrated increased signal intensity involving the proximal terminal branches of the left brachial plexus at the retropectoralis minor space. No signal abnormalities or structural compression were identified within the interscalene triangle or costoclavicular interval, supporting isolated compression beneath the pectoralis minor tendon (Figure 1).

After completing the diagnostic work-up, the need for intervention was discussed with the patient, who accepted and underwent a tenotomy of the pectoralis minor and a release and neurolysis of the suprascapular nerve (SSN) by shoulder arthroscopy.

Surgical technique

The surgery was performed in the beach-chair position with the patient under a preoperative interscalene brachial plexus block and general anesthesia. Standard arthroscopic equipment was used, including a 30-degree arthroscope, an arthroscopic shaver, and an arthroscopic radiofrequency ablation device.

After a standard diagnostic arthroscopy, the arthroscope was placed in the lateral subacromial portal. The anterior bursal tissues were removed with a shaver or radiofrequency ablation device in the space between the anterior deltoid and the subscapularis in order to visualize the lateral aspect of the coracoid and the lateral conjoined tendon.

An anterior working portal was then established, followed by release of the rotator interval, allowing visualization of the intact subscapularis tendon. An accessory Neviaser portal was established, and then a Wissinger was placed over it to always protect the suprascapular artery (SSA) to continue releasing the surrounding soft tissues with radiofrequency until observing the transverse suprascapular ligament (TSL), which was resected with the help of arthroscopic scissors due to the compression of the SSN, which showed edema and perineural fibrosis. Adequate release of the SSN was observed afterward. Care was taken to avoid injury to the brachial plexus and vessels and to identify any abnormal adhesions.

**Figure 2:**
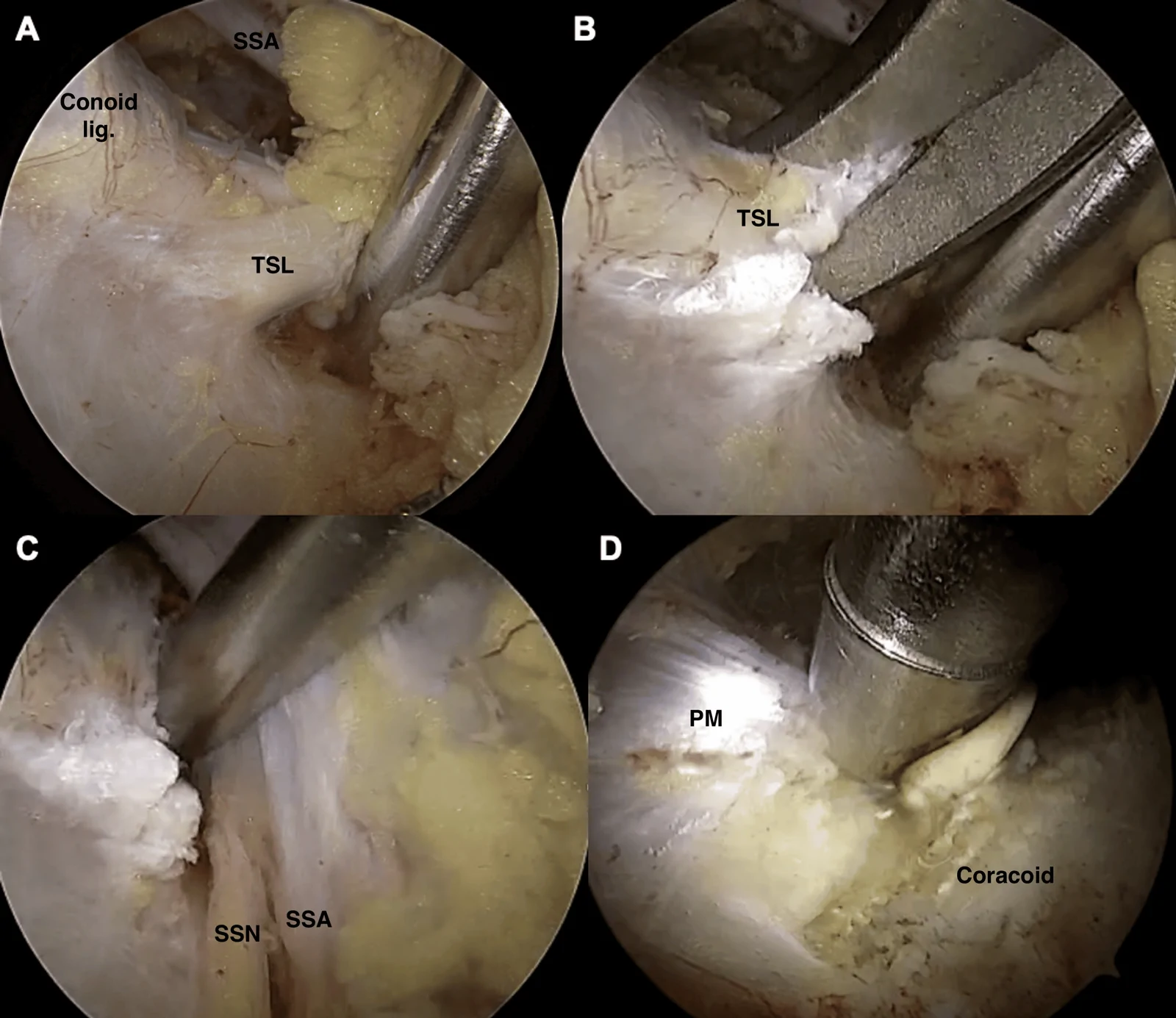
Arthroscopic release of the pectoralis minor. (A) Identification of the SSA, TSL, and conoid ligament. (B) Release of the TSL. (C) Release of the SSN. (D) Detachment of the PM. SSA: suprascapular artery; TSL: transverse suprascapular ligament; SSN: suprascapular nerve; PM: pectoralis minor.

Next, a radiofrequency ablation device was used to detach the tendon of the pectoralis minor muscle subperiosteally from the medial aspect of the coracoid with the thermal end facing superiorly and laterally. Complete release of the pectoralis minor tendon with medial retraction of the released muscle was confirmed under direct visualization. Figure 2 illustrates the main surgical steps. Figure 2A shows identification of SSA, TSL, SSN, and conoid ligament. Figure 2B demonstrates release of the TSL. Figure 2C shows decompression of the SSN, whereas Figure 2D demonstrates arthroscopic detachment of the pectoralis minor tendon from the medial aspect of the coracoid. There were no intraoperative or postoperative complications.

Outcomes

After arthroscopic release of the pectoralis minor tendon, the patient was immobilized in a sling after surgery. Sutures were removed at week two postoperatively, and physical therapy exercises were initiated for motion and strength. At 12 months, there was a significant change in the DASH and Constant-Murley scores, 19.2% and 79%, respectively. Pain intensity improved substantially, with the VAS score decreasing from 9 preoperatively to 2 at the 12-month follow-up; the patient underwent physical therapy, which improved muscle atrophy in the supraspinatus and infraspinatus muscles. The patient reported high satisfaction with the surgical outcome after she had undergone two major spinal surgeries that had failed to improve her condition and had only caused her symptoms to worsen. One year after the surgery, she regained the quality of life she desired and returned to the recreational activities she had put aside because of the constant pain she was experiencing.

## Discussion

Our patient presented with radiographic evidence of multilevel cervical degenerative disc disease, congenital cervical canal narrowing at C4-C5, C5-C6, and C6-C7, and bilateral neural foraminal stenosis, predominantly on the left side. These findings appropriately supported the indication for anterior cervical discectomy and fusion followed by left C6-C7 laminectomy, foraminotomy, and decompression because of persistent radicular symptoms. Nevertheless, the complete absence of clinical improvement after both technically successful procedures, together with the characteristic provocative examination findings and brachial plexus MRI abnormalities localized to the retropectoralis minor space, strongly suggested that isolated PMS represented the principal source of the patient's persistent symptoms. Although concomitant cervical spondylotic disease cannot be completely excluded, the marked postoperative improvement following arthroscopic pectoralis minor release supports PMS as the predominant pain generator in this case.

Recognition of PMS has increased [12] in recent years as understanding of dynamic compression around the coracoid process has evolved. The principal provocative maneuvers and complementary diagnostic studies currently used in the evaluation of isolated PMS are summarized in Table 1. Current evidence indicates that compression beneath the pectoralis minor tendon should be considered separately from other forms of neurogenic thoracic outlet syndrome [12] because the mechanism of compression, clinical examination, and surgical management may differ. Awareness of this condition is particularly important in patients whose symptoms cannot be explained by cervical imaging or routine shoulder evaluation.

Establishing the diagnosis remains challenging because no individual clinical test or imaging study provides definitive confirmation. Instead, diagnosis depends on integrating the patient's history with physical examination findings, provocative maneuvers, and complementary imaging studies. Although ultrasound-guided pectoralis minor blocks [13] have been proposed as useful diagnostic adjuncts, they are not mandatory in every patient. In our patient, magnetic resonance imaging performed on a high-resolution 1.5-T scanner demonstrated signal abnormalities adjacent to the brachial plexus, providing valuable complementary information after cervical pathology had been treated but persistent symptoms remained unexplained by the postoperative cervical evaluation.

Surgical treatment was considered after prolonged failure of conservative management and persistence of disabling neurological symptoms. Arthroscopic release offered the advantage [14] of direct visualization of the neurovascular structures while minimizing soft-tissue disruption. This minimally invasive approach also allowed simultaneous decompression of the suprascapular nerve, providing comprehensive treatment of the identified compression sites during a single procedure.

Our postoperative evolution parallels the favorable outcomes described in recent arthroscopic series [14]. The patient experienced marked pain reduction together with substantial improvement in functional outcome scores and return to recreational activities during follow-up. Historical series have also demonstrated favorable outcomes following isolated pectoralis minor tenotomy, even before the widespread adoption of arthroscopic techniques [15]. Although this report represents a single clinical observation, the results support previously published evidence indicating that arthroscopic pectoralis minor release can be an effective option for carefully selected patients [16] with isolated PMS after unsuccessful conservative treatment.

Our findings are also consistent with the evolution of arthroscopic techniques described in recent literature. Wagner et al. further refined minimally invasive infraclavicular brachial plexus decompression by emphasizing safe identification of neurovascular structures and reproducible arthroscopic access to the retropectoralis minor space, supporting arthroscopy as an effective approach for carefully selected patients with PMS and neurogenic thoracic outlet syndrome [17].

Despite the satisfactory outcome, this report has several limitations. First, it describes the clinical course of a single patient, limiting the generalizability of the findings. Second, electrodiagnostic studies, including nerve conduction studies and electromyography, were not performed; therefore, concomitant cervical radiculopathy or peripheral neuropathy cannot be completely excluded. Nevertheless, the combination of persistent symptoms despite two appropriately indicated cervical spine procedures, characteristic provocative physical examination findings, brachial plexus MRI abnormalities localized to the retropectoralis minor space, and the patient's marked clinical improvement following arthroscopic pectoralis minor release strongly supports isolated PMS as the principal source of her persistent symptoms. Future prospective studies including larger patient cohorts, standardized outcome measures, and longer follow-up periods are necessary to further define patient selection criteria and long-term outcomes following arthroscopic pectoralis minor release.

## Conclusions

PMS should be considered in patients with persistent upper-extremity pain, paresthesia, or weakness despite appropriate treatment for cervical spine disorders. Careful history taking, targeted physical examination, provocative maneuvers, and complementary imaging are essential to avoid delayed diagnosis and unnecessary procedures. In selected patients who fail conservative management, arthroscopic pectoralis minor release with brachial plexus neurolysis represents a safe and effective minimally invasive option that can provide substantial clinical improvement when performed by experienced shoulder surgeons. Early recognition of this uncommon condition may prevent unnecessary cervical spine surgery and substantially improve patient outcomes.
